# Managing patient flows in radiation oncology during the COVID-19 pandemic: Reworking existing treatment designs to prevent infections at a German hot spot area University Hospital

**DOI:** 10.1007/s00066-020-01698-6

**Published:** 2020-10-29

**Authors:** Dennis Akuamoa-Boateng, Simone Wegen, Justin Ferdinandus, Regina Marksteder, Christian Baues, Simone Marnitz

**Affiliations:** 1 Department of Radiation Oncology, Center for Integrated Oncology, University Hospital Cologne, Cologne, Germany; 2 Department of Nuclear Medicine, University Hospital Essen, Essen, Germany; 3 Department of Hospital Pharmacy, University Hospital Cologne, Cologne, Germany

**Keywords:** COVID-19, SARS-CoV-2, Radiation oncology, Disease transmission, infectious, Radiation dose hypofractionation, Stereotactic radiosurgery

## Abstract

**Purpose:**

The described work aimed to avoid cancellations of indispensable treatments by implementing active patient flow management practices and optimizing infrastructure utilization in the radiation oncology department of a large university hospital and regional COVID-19 treatment center close to the first German SARS-CoV‑2 hotspot region Heinsberg in order to prevent nosocomial infections in patients and personnel during the pandemic.

**Patients and methods:**

The study comprised year-to-date intervention analyses of in- and outpatient key procedures, machine occupancy, and no-show rates in calendar weeks 12 to 19 of 2019 and 2020 to evaluate effects of active patient flow management while monitoring nosocomial COVID-19 infections.

**Results:**

Active patient flow management helped to maintain first-visit appointment compliance above 85.5%. A slight appointment reduction of 10.3% daily (*p* = 0.004) could still significantly increase downstream planning CT scheduling (*p* = 0.00001) and performance (*p* = 0.0001), resulting in an absolute 20.1% (*p* = 0.009) increment of CT performance while avoiding overbooking practices. Daily treatment start was significantly increased by an absolute value of 18.5% (*p* = 0.026). Hypofractionation and acceleration were significantly increased (*p* = 0.0043). Integrating strict testing guidelines, a distancing regimen for staff and patients, hygiene regulations, and precise appointment scheduling, no SARS-CoV‑2 infection in 164 tested radiation oncology service inpatients was observed.

**Conclusion:**

In times of reduced medical infrastructure capacities and resources, controlling infrastructural time per patient as well as optimizing facility utilization and personnel workload during treatment evaluation, planning, and irradiation can help to improve appointment compliance and quality management. Avoiding recurrent and preventable exposure to healthcare infrastructure has potential health benefits and might avert cross infections during the pandemic. Active patient flow management in high-risk COVID-19 regions can help Radiation Oncologists to continue and initiate treatments safely, instead of cancelling and deferring indicated therapies.

## Introduction

The continued global spread of the severe acute respiratory syndrome coronavirus 2 (SARS-CoV-2) and the associated risk of pulmonary manifestations of coronavirus disease-19 (COVID-19) posed a challenge to all human societies in late 2019 and early 2020 [19]. Primarily, three horizontal transmission pathways are discussed which have an impact on the contact guidelines from the Societies of Radiation Oncology [20, 21]: droplet infection, contact infection, and airborne transmission [11, 13, 22]. In the absence of targeted treatment options such as specific antiviral medication or vaccines, primary prevention in the form of isolation, quarantine, social distancing, and community containment have been key response mechanisms to control the pandemic [17]. Due to the late symptom onset alongside high numbers of asymptomatic manifestations, it has been widely reported that controlling viral transmission is crucial to reduce nosocomial disease spread between personnel and patients, especially in healthcare facilities [23].

As a population at risk during the ongoing pandemic [24], cancer patients and their healthcare providers must constantly balance the risks of SARS-CoV‑2 infection associated with diagnostic or therapeutic procedures against the risk of a potential delay in treatment, while medical infrastructure capacities and medical resources are reduced. For patients undergoing external beam radiation therapy (EBRT), most regimens are fractionated and require sequential visits. Healthcare providers globally need to adapt their patient flows and reorganize treatment pathways to continue indispensable treatments. At the University Hospital of Cologne, Germany, the outpatient admission and treatment processes were remodeled. Active patient flow management was implemented early, at the onset of the pandemic, to adapt to the potential shortage of staff, supply, and government-regulated reduction in hospital treatment capacity [15, 25] instead of cancelling indicated therapies.

In 2019, a total of 2174 patients were treated in the Department of Radiation Oncology of the University Hospital of Cologne, of whom 52% were male and 48% female. The University Hospital of Cologne is a maximum medical care provider and regional COVID-19 treatment center at the center of Europe, located close to Germany’s first pandemic hotspot in Heinsberg. Generating about €10.61 million in earnings before interest and taxes (EBIT) of over €1.08 billion^1^ combined annual turnover in 2018, with 58 departments and 1540 beds, a total number of 360,882 patients—62,862 inpatients and 321,585 outpatients—were treated by 10,700 staff members in 2018 [26].

We here report on our experiences on managing the early COVID-19 pandemic, before actionable recommendations of the respective Radiation Oncology Societies were published.

## Patients and methods

As radiation oncology treatment planning requires multidisciplinary interactions across treatment teams, efforts were put into upscaling IT capacities. Telemedical cancer board meetings via videocall options were implemented and essential meetings were limited to five persons. Regular daily morning, noon, and afternoon physician meetings were replaced by phone and secure digital communication. Home office work was implemented for contouring and treatment planning. Telemedical appointments were offered during and after radiation treatment.

To abide by strict official disinfection [9], hygiene [27], contact, and distancing regulations at the University Hospital of Cologne, personnel and patients wear a face mask across the entire campus and must keep a minimal distance of 1.5 meters to each individuum.

Shift fluctuation of personnel was reduced to secure regular personnel and patient setups by long-term shift assignment and reduced cross-team personnel fluctuation. A strict ban on visitors was put in place for both the outpatient and inpatient departments. Exceptions were individually discussed and mainly applied to legal medical attendants.

All sorts of business trips were cancelled and personnel with transnational travelling history were required to abide by a 14‑day campus access prohibition ruling. Staff showing symptoms of upper respiratory tract viral infection were prohibited from entering the campus until 48 h after reconvalescence alongside negative SARS-CoV‑2 PCR testing.

Appointments were individually rescheduled to reduce peaks of patients by separating patient flows and reclassifying severity groups. All treatments of benign diseases were pretermitted. Curative, definitive, and palliative radiation treatment regimens were prioritized over postoperative adjuvant treatments. Protocols were performed according to standard of care practices, preferring hypofractionation where applicable.

Overbooking of appointments to control for potential patient no-shows was suspended to increase patient and staff safety. Instead, reminders were implemented by actively calling patients prior to all types of appointments. To increase departments’ efficiency while simultaneously reducing the number of patients in common waiting spaces, patients were urged to wait outside the facility and allowed to enter the facility just shortly before any appointment using strict individual timeslots. To reduce the time per patient spent in the facility, patients were grouped into four time categories A–D of short, medium, long, and extra-long expected planning CT timeslots, respectively, to control the time per patient and to ensure time to comply with hygiene and disinfection guidelines.

Prescreening of patients’ full-track records reduced the number of multiple appointments and helped in assigning patients to severity groups A–D, estimating the potential time spent within the facility and the case complexity for treatment discussion, planning, and radiation sessions.

Every inpatient received a COVID-19 PCR test on the day of admission and was required to hold a negative test result not older than 72 h at the beginning of every elective invasive medical procedure, such as fiducial, port, intrauterine device, or feeding tube implantation. This routine did not apply to interventions or operations whose medical urgency did not tolerate any delay due to awaiting negative test results. In this case, special hygiene protocols had to be applied to secure safe operation and prevent asymptomatic viral spread.

A detailed post-interventional analysis of the outpatient working routines was performed, totaling a 37-working day observation period of 2019 and 2020 for calendar weeks 12–19 year-to-date. From the inpatient clinic, analyses via PCR testing were conducted for every hospital admission dating from March 15 to May 7, 2020.

For comparison, datasets used in this study cover the periods from March 18 to May 10, 2019, and March 16, to May 08, 2020, encompassing outpatient care of publicly and privately insured patients.

Statistical analyses of two-sided *t*-tests were performed by the authors using Microsoft Excel Office 16 (Redmond, WA, USA) and R version 3.5.0 released 23.04.2018 (R Foundation for Statistical Computing, Vienna, Austria). A *p*-value of less than 0.05 was considered statistically significant.

## Results

Changing workflow designs and patient selection led to reduced first-contact appointments and significantly increased downstream appointment compliance. For the observed periods, the daily number of first-contact, publicly insured patients scheduled ([confidence interval, CI, 95%; standard deviation, SD, 1.0 vs. 1.3] 7.1 vs. 6.4; *p* = 0.004) and presenting ([CI 95%; SD 1.4 vs. 1.3] 6.3 vs. 5.4; *p* = 0.0024) to the outpatient clinic was significantly lower in 2020 compared to 2019. We observed a 10.3% reduced scheduling rate to 89.7% for 2020 appointments compared to 2019, and a 14.3% reduction of patient presentation to 85.7%. However, there was no significant difference in the overall show rate (88.9% vs. 85.5%; *p* = 0.165; Table 1).

**Table 1:** Patient flow management figures of 2019 and 2020

	2019		2020	
	Total number	Daily number	Total number	Daily number
Public ambulance scheduled	262	7.08	235	6.35
Public ambulance presented	233	6.30	199	5.80
Show rate public ambulance	88.93%	–	84.68%	–
Private ambulance scheduled	99	2.70	105	2.84
Privat ambulance presented	91	2.46	92	2.49
Show rate private ambulance	91.92%	–	87.62%	–
Planning CT scheduled	346	9.35	394	10.68
Planning CT performed	305	8.24	369	9.97
Show rate CT	88.15%	–	93.65%	–
Treatment started	276	7.46	327	8.84
Aftercare clinic public	318	12.23	6	0.16
Aftercare clinic private	48	2.08	13	0.35
CyberKnife treatment start	48	1.45	67	2.23
CyberKnife treatments	105	3.18	99	3.3

CyberKnife: Accuray, Sunnyvale, CA, USA

For the private outpatient clinic there was no difference evident for daily scheduled ([CI 95%; SD 1.4 vs. 1.1] 2.7 vs. 2.8, *p* = 0.331) and presenting ([CI 95%; SD 1.4 vs. 0.1] 2.5 vs. 2.5, *p* = 0.468) patients. Notably, we observed slight increases for scheduled (6.1%) and presented patients (1.1%) compared to 2019 appointments.

Patient prioritization by disease severity classification led to significantly higher numbers of daily scheduled ([CI 95%, SD 1.4 vs. 1.6] 10.6 vs. 9.4, *p* = 0.0001) and performed ([CI 95%; SD 1.4 vs. 1.6] 10.0 vs. 8.2, *p* = 0.0001) planning CTs in 2020. The daily patient show rate was significantly increased from 88.2% in 2019 to 93.7% in 2020 (*p* = 0.009). Overall, we observed a 13.9% increase in CTs scheduled and a 20.1% increase in CTs performed for 2020 compared to the 2019 baseline.

While appointments were handled more restrictively, the daily number of patients starting radiation treatment was still significantly increased from 2019 to 2020 ([CI 95%; SD 2.93 vs. 0.63] 7.5 vs. 8.8, *p* = 0.026) by 18.5%.

Hypofractionation and acceleration regimes were frequently used. Reducing the timespan under therapy per patient, we observe significantly increased numbers of daily stereotactic radiosurgery (SRS) treatment initiations ([CI 95%, SD 1.20 vs. 1.14] 1.45 vs. 2.23; *p* = 0.0043). This leads to an increase from 45.71% (48/105) to 67.68% (67/99) of first treatments of all fractions per day, while no significant differences (*p* = 0.364) were observed in the daily average number of treated patients using the CyberKnife (Accuray, Sunnyvale, CA, USA; [CI 95%, SD 1.53 vs. 1.14] 3.18 vs. 3.3).

The number of patients presenting to the aftercare clinic was reduced for both the public (318 vs. 6, *p* = 0.0001) and the private (48 vs 13 *p* = 0.001) sector. Despite expectable financial losses, daily presentation was reduced from an average of 12.2 in 2019 to 0.2 for the public and 2.1 to 0.4 patients for the private sector.

Restructuring key processes helped to continue and initiate treatments safely. Out of a total of 913 hospital bed days, patients spent on average 5.67 days (median 3; SD 6.72) on the ward. Testing a total number of 74 patients or all individual 164 in-hospital cases from March 15 to May 07, no positive RT-PCR test result for any probe analyzed was observed.

## Discussion

Healthcare providers must increasingly integrate supply chain management routines into their workflows. Quality management and prediction can drive hospital efficiency, care provider productivity, and patient satisfaction [10].

During the SARS-CoV‑2 pandemic, cancer patients form a major risk group for severe complications [3, 24]. Oncologists must weigh up potential risks of COVID-19 morbidity and mortality against the advantages of intended therapies [5], as delaying potentially curative treatments affects oncologic outcomes. The vast majority of 95% of surgeons believe that timely multimodal treatments should be performed according to standard therapy indications for colorectal carcinomas despite the COVID-19 pandemic [1]. For every month of radiotherapy deferral, head and neck cancer patients’ mortality risk increases by 16%, and a 4‑week delay in adjuvant chemotherapy for colorectal and breast cancer is associated with poorer overall survival [5]. Rather than cancelling indicated treatments, the current authors implemented new workflow designs managing to initiate and continue treatments safely.

Underutilization of medical resources has negative impacts by increasing healthcare costs, decreasing access to care, and reducing efficiency and productivity of care providers [7]. As shown in this study, it is possible to remodel the CT program. Despite reducing the number of patients, it was possible to significantly increase the number of CTs scheduled by 20.1% (*p* = 0.0001) and performed by 13.9% (*p* = 0.0001), whilst controlling the time of CT machine occupation per patient during the whole shift.

The most common reasons for missing medical appointments are known to be forgetting (35.5%) and miscommunication (31.5%) [28]. Therefore, it is elsewhere recommended to proactively schedule patients to diminish negative impacts of patient no-shows [8]. While predictive models propose overbooking approaches to significantly reduce patient waiting by at least 6%, 27% on overtime, and 3% on total costs compared to flat-overbooking methods [7], the authors’ department focused early on actively controlling appointment compliance while avoiding overbooking. It has been published elsewhere that pre-appointment reminder calls can effectively decrease no-show rates by 19% [12]. As the implemented process of active communication is time consuming, future processes should include automation procedures for reminders. Still, having been forced to immediately react to the pandemic and quickly redesign key processes, the authors have implemented an actionable system with the resources available. In general healthcare, staff reminder calls can reduce no-show rates from 23.1 to 13.6% [14] and nonreceipt of appointment reminders 2 h before appointments strongly correlates with 15- to 60-minute tardiness (*p* < 0.0001), >60-minute tardiness (*p* < 0.0001), and no-shows (*p* < 0.0001) [19]. The aforementioned interventions were reported to significantly reduce no-show rates of 29.2% to as low as 22.8% (*p* < 0.001), while cancellation (13.1% vs. 11.5%, *p* = 0.15) and rescheduling rates (14.2% vs. 12.2%, *p* = 0.09) can be insignificantly reduced, which is similar to our findings. During the pandemic, active calling helped to ensure radiation treatment initiation and continuation, alleviating patients’ anxiety and insecurity.

Patients with deferrable treatments were rescheduled after having actively been discussed in interdisciplinary cancer board meetings. Additionally, external patients were referred to near-to-home facilities. Due to this changed patient selection and reduction of multiple appointments, the authors found that while reducing the overall number of first appointments slightly by 10.3%, the resulting daily rate of radiation therapy initiation was still increased by 18.5% (*p* = 0.026).

Non-treatment-related routine follow-up appointments were deferred in mutual agreement with patients and rescheduled within 2 to 4 months in close consultation with the primary oncology care giver. Routine follow-up imaging procedures were recommended and performed after individual case discussions. However, exceptions were made for the first aftercare appointment and prioritized via telemedical infrastructure. Hereby, the overall daily number of patients presenting to the aftercare clinic was significantly reduced for both the public (*p* < 0.001) and the private sector (*p* = 0.001), saving resources for immediate cancer care.

The authors’ department performed accelerated and hypofractionated schemes for established treatment protocols more frequently, leading to increasing SRS initiation rates from 45.71 to 67.68% of first treatments of all fractions daily. This led to a reduced time per patient for completion of the desired treatment and was later recommended by Radiation Oncology Specialist Societies [4, 15, 18, 20, 29, 30], for example for bone metastases (1 × 8 Gy and 5 × 4 Gy) and mild simultaneous integrated boost (SIB) hypofractionation for localized prostate cancer (pT1b–T3aN0M0), analogously to the phase III CHHip trial [2].

The primary transmission mode of SARS-CoV‑2 is described as human to human [16]. It was reported that 1080 health workers in Wuhan were infected and among 138 hospitalized patients diagnosed with COVID-19, 41% were suspects of nosocomial transmission, resulting in 26% intensive care unit treatments and a mortality rate of 4.3%. To reduce potential nosocomial infections, the authors’ department aimed to avoid patient clustering, as stochastic models have identified the efficacy of reducing inter-personnel contacts [6].

Despite dedicated testing of all inpatients, neither positive tests for staff nor for patients were observed, while exploratory analyses of the first 72,000 cases of COVID-19 in China report 3.8% of cases detected among healthcare personnel, leading to a 0.3% death rate of healthcare workers. Of all cases reported, only 0.5% of patients showed malignancies as comorbidities [31]. Not detecting positive RT-PCRs for the current inpatient cohort could potentially be attributed to the relatively low overall prevalence in Germany [32]. However, it has been described that false-negative test results can occur due to test and sampling errors [33]. Despite this, the lack of observed nosocomial infections among patients and personnel and the absence of symptoms of acute respiratory distress syndrome (ARDS) within an explicit German hotspot area and regional COVID-19 treatment center lead the authors to have confidence in the true nature of the results. In addition, the urge to treat a patient with positive test results was not experienced. However, alternative active patient flow management procedures were prepared by installing a hermetically sealed infrastructure and exclusively assigned personnel governed by security concepts. These mechanisms are not subject of this analysis.

## Conclusion

During the upcoming period of recovery and increasing number of treatment facility reopenings within healthcare services, radiation oncologists will be challenged with pivotal process reconsiderations. Implementing reshaped workflows within regular medical routines might be fundamental to meeting the demand of patients accumulated during the healthcare system lockdown. Precise scheduling and appointment communication amidst a period of decreased institutional capacities can help to reduce no-show rates while avoiding overbooking practices. Streamlined patient flows allow reduction of the time spent within the facility. Preferring accelerated hypofractionated treatments over normofractionated regimes can reduce machine occupancy rates and effectively help to treat more patients in less time. As the COVID-19 pandemic is still ongoing, it will be a top priority to design actionable workflows that can best prevent nosocomial infections of patients and personnel to safely continue and start radiation instead of cancelling or deferring indicated therapies.

Further investigation should be performed to identify noninferior treatment regimes to hypofractionate and accelerate radiation fractionation schedules and hence reduce overall facility time per patient and the associated financial impact. Restructuring key processes using automation might be beneficial for healthcare providers to implement adapted patient flow management into future medical routines.

## Footnotes

1 American “billion” that is identical to the German “Milliarde“.
