# Correction to: Managing patient flows in radiation oncology during the COVID-19 pandemic: Reworking existing treatment designs to prevent infections at a German hot spot area University Hospital

**DOI:** 10.1007/s00066-020-01724-7

**Published:** 2020-12-15

**Authors:** Dennis Akuamoa-Boateng, Simone Wegen, Justin Ferdinandus, Regina Marksteder, Christian Baues, Simone Marnitz

**Affiliations:** 1 Department of Radiation Oncology, Center for Integrated Oncology, University Hospital Cologne, Cologne, Germany; 2 Department of Nuclear Medicine, University Hospital Essen, Essen, Germany; 3 Department of Hospital Pharmacy, University Hospital Cologne, Cologne, Germany

**Correction to:**

**Strahlenther Onkol 2020**

10.1007/s00066-020-01698-6

The original version of this article unfortunately contained a mistake.

Section “Patients and methods,” fourth paragraph, first sentence, should read: All sorts of business trips were cancelled and personnel with transnational travelling history were required to abide by a 14-day campus access prohibition ruling.

The original article has been corrected.

